# Facility-Level Variation in Major Leg Amputation Among Patients With Newly Diagnosed Diabetic Foot Ulcer

**DOI:** 10.1001/jamanetworkopen.2025.6781

**Published:** 2025-04-23

**Authors:** Hiroyuki Suzuki, Mary Vaughan-Sarrazin, Michael Ohl, Bradley Mecham, Kimberly McCoy, Meghan B. Brennan, Jeffrey M. Robbins, Daniel J. Livorsi

**Affiliations:** 1 Veterans Rural Health Resource Center–Iowa City, Veterans Affairs (VA) Office of Rural Health, Iowa City, Iowa; 2 Center for Access & Delivery Research and Evaluation, Iowa City VA Health Care System, Iowa City, Iowa; 3 Department of Internal Medicine, University of Iowa, Iowa City; 4 Department of Medicine, School of Medicine and Public Health, University of Wisconsin, Madison; 5 William S. Middleton Memorial VA Medical Center, Madison, Wisconsin; 6 Specialty Care Services, VA Central Office, Washington, DC

## Abstract

**Importance:**

The prevalence of diabetes is increasing over time, fueling an epidemic of diabetic foot ulcers (DFUs) and subsequent risk of leg amputation. However, little is known about the variation in outcomes for patients with DFUs according to the health care facilities treating them.

**Objective:**

To examine facility-level variation in major leg amputation among veterans with incident DFUs using the Veterans Health Administration (VHA) cohort.

**Design, Setting, and Participants:**

A retrospective cohort study was conducted from January 1, 2016, to December 31, 2021, of all veterans with a new diagnosis of DFU at 140 VHA facilities across the US. Patients were followed up to 1 year from DFU diagnosis. Analyses were conducted between March 22, 2024, and January 13, 2025.

**Exposure:**

A facility was assigned to each patient corresponding to the health care site where the initial DFU diagnosis was made.

**Main Outcomes and Measures:**

The primary outcome was major leg amputation during the follow-up period. A multivariable mixed-effects regression model with random facility intercepts was applied to assess variation in major leg amputation rates across facilities, adjusting for social drivers of health, comorbidities, and complicated DFU at initial diagnosis. The median odds ratio (MOR) was calculated to quantify facility-level variation in outcomes.

**Results:**

A total of 86 094 veterans (98.3% male; mean [SD] age, 73.0 [8.1] years; age range, 55-102 years) were included. Major leg amputation was performed for 3279 veterans (3.8%) within a year of DFU diagnosis. The MOR for facility-level variation in major leg amputation was 1.85, indicating that the odds of major leg amputation were 1.85 times higher between 2 randomly selected facilities for an average patient (*P* < .001). In contrast, the MOR for facility-level variation in 1-year mortality was 1.16 (*P* < .001).

**Conclusions and Relevance:**

This cohort study of veterans with newly diagnosed DFU found significant facility-level variation in major leg amputation rates within 1 year of DFU diagnosis. Facility-level variation in 1-year mortality rates was much smaller, suggesting variation in leg amputation was likely to stem from variation in DFU-specific care. The VHA should strive to minimize the odds of major leg amputation and interfacility variation.

## Key Points

**Question:**

How much facility-level variation in major leg amputation rates exists among veterans with newly diagnosed diabetic foot ulcers?

**Findings:**

In this 6-year cohort study of 86 094 veterans newly diagnosed with diabetic foot ulcers at 140 Veterans Affairs facilities, the odds of major leg amputation were 1.85 times higher between 2 randomly selected facilities for an average patient. The facilities’ odds ratio for facility-level variation in major leg amputation, adjusted for all social drivers and patient-level comorbidities, ranged from 0.29 to 3.53, with facility-level variation in the major leg amputation much larger than facility-level variation in 1-year mortality.

**Meaning:**

These findings suggest that facility-level variation, likely related to diabetic foot ulcer–specific care, might be an important driver of variation in major leg amputation.

## Teaser

This cohort study examines facility-level variation in leg amputation rates among veterans newly diagnosed with diabetic foot ulcers.

## Introduction

Diabetes affects approximately 10% of adults in the US, with rates increasing to 25% among veterans.^1,2^ The prevalence of diabetes in both adults and veterans is increasing over time, fueling an epidemic of diabetic foot ulcers (DFUs) and subsequent risk of leg amputations. Indeed, hospitalizations due to leg amputation significantly increased from 2010 to 2020.^3^ In addition to the human toll, the financial impact is substantial. Costs are 5 times higher for patients after a major (above or below knee) amputation compared with those with diabetes alone.^4,5^

US patients with diabetes shoulder the burden of leg amputation unequally. Outcomes for patients with DFU vary by race, ethnicity, and geographic region.^6,7,8,9,10,11,12,13^ These studies suggest that variations in leg amputation risk may be associated with social drivers of health through complex interactions among biological, behavioral, environmental, and health care system factors.^14^ The health care system factor presents a tangible point of potential intervention for the medical field. However, little is known about the variation in outcomes for patients with DFU related to the health care facilities treating them. In patients undergoing peripheral vascular intervention for critical limb ischemia, Provance et al^15^ reported that the variation in 30-day leg amputation rates across 179 US health care sites was more strongly explained by hospital-level rather than patient-level factors. However, it is unclear whether this finding is generalizable to other settings. If variation in leg amputation is found for patients with DFU, health service–based interventions might be particularly impactful for standardizing high-quality care across facilities and potentially reducing disparities by offsetting social drivers of health.

Health care systems should strive for both excellence and equity. For DFU care, this means minimizing the risk of leg amputation as well as the variation of that risk across facilities. The Veterans Health Administration (VHA) is the largest integrated health care system in the US, providing care for more than 9 million veterans at 1380 health care sites annually. The system provides an opportunity to examine facility-level variation in outcomes for veterans with DFU when barriers related to insurance and access are relatively small. In this study, we used national VHA administrative data to examine facility-level variation in leg amputations among veterans with incident DFUs. Measuring how much variation is attributable to facility-level factors provides an estimate of the extent to which limb salvage could be improved by standardizing high-quality care throughout the nation’s largest health care system.

## Methods

### Study Design, Database, and Definitions

This retrospective cohort study assessed all adult veterans with a new diagnosis of DFU within the VHA from January 1, 2016, to December 31, 2021. This study was approved by the institutional review boards of the University of Iowa and the Iowa City Veterans Affairs (VA) Health Care System Research and Development Committee. These institutional review boards waived informed consent because the study involves secondary data analysis and no more than minimal risk to participants. The study followed the Strengthening the Reporting of Observational Studies in Epidemiology (STROBE) reporting guideline.^16^

We obtained data from the VHA Corporate Data Warehouse, a national repository that aggregates clinical and administrative records from VA health care facilities.^17^ Veterans with DFU were identified using validated *International Statistical Classification of Diseases and Related Health Problems, Tenth Revision* (*ICD-10*) diagnostic codes (eTable 1 in Supplement 1).^6,18,19,20^ To ensure reliability, we excluded data recorded during encounters with physicians or nurses unlikely to be directly involved in DFU care (ophthalmology, dentistry, and mental health). We also excluded veterans who had an *International Classification of Diseases, Ninth Revision* (*ICD-9*) or *ICD-10* code for DFU in 2015 to ensure that only new DFU diagnoses were included in the study. Additional variables for risk adjustment included demographics (age, sex, race, and ethnicity), rurality, drive time to nearest VHA tertiary hospital, comorbidities selected from the Charlson Comorbidity Index and Elixhauser Comorbidity Index,^21^ and complicated DFU at presentation. Those variables were chosen because they have been reported to be associated with major leg amputations in previous studies.^5,6,18,19^ Race (Asian, Black, American Indian or Alaska Native, Native Hawaiian, Pacific Islander, White, multiracial, or unknown) and ethnicity (Hispanic or Latino, not Hispanic or Latino, or unknown) were predominantly self-reported or reported by proxies and stored in the VHA Corporate Data Warehouse. Veterans were classified as having a complicated DFU if they had an *ICD-10* code for osteomyelitis or gangrene within 2 weeks before or 1 week after the DFU diagnosis (eTable 1 in Supplement 1). Veterans without these codes were classified as having an uncomplicated DFU. The Social Vulnerability Index (SVI) from the Centers for Disease Control and Prevention^22^ was used as an area-level measure of socioeconomic disadvantage and linked to patient records by the census tract of veteran residence. Rurality was defined as urban or rural/highly rural as determined by the geocoded location of the home of each veteran.^23^ Within the VHA system, a small number of hospitals and affiliated clinics form a regional health care system considered as a single facility. We assigned each veteran to the facility corresponding to the health care site where they received their initial DFU diagnosis. The drive time from the veteran’s home to the nearest VHA tertiary hospital was determined using data obtained from the Planning Systems and Support Group files. The primary outcome was a major leg amputation within 1 year of the DFU diagnosis. Major leg amputation was identified using *ICD-10* codes and *Current Procedure Terminology* (*CPT*) codes (eTable 1 in Supplement 1).^6,24^ The secondary outcome was all-cause mortality within 1 year of the DFU diagnosis. We obtained mortality dates from the VHA Vital Status File, which has excellent agreement with the National Death Index.^25^ We contrasted facility-level variation in major leg amputation with that in all-cause mortality, aiming to assess disparities in care specifically related to DFU.

### Statistical Analysis

Analyses were conducted between March 22, 2024, and January 13, 2025. Descriptive statistics for patient characteristics and outcomes were calculated as numbers and percentages. Comparisons between veterans who had major leg amputation and those who did not were assessed using standardized mean differences.^26^ We applied a multivariable mixed-effects regression model with random facility intercepts to assess variation in major leg amputation rates across VHA facilities, adjusting for demographics, comorbidities, and the complicated DFU at initial diagnosis. We estimated odds ratios (ORs) and 95% CIs to assess the associations between patient-level variables and major leg amputation. By exponentiating random facility intercepts, we derived the ORs and 95% CIs for major leg amputation in each facility relative to the average facility. We also calculated the median odds ratio (MOR) to quantify facility-level variation in outcomes.^27^ The MOR represents the median change in odds of major leg amputation when comparing care between 2 randomly selected facilities while holding patient-level variables constant. For example, an MOR of 1.0 indicates no variation in outcome by facility, whereas an MOR of 1.2 indicates that 2 identical hypothetical patients would have a 20% difference in the odds of major leg amputation if treated in one random facility vs another.^15^ To evaluate whether the addition of random facility intercepts, representing facility variation, significantly improved model fit, we performed a likelihood ratio test comparing models with and without the facility-level random intercepts. If the addition of random facility intercepts significantly improves the model, it suggests that there is a significant facility-level variation in major leg amputation. We also evaluated how patient characteristics affected major leg amputation rates and how much of the facility-level variation in rates remained by comparing the MOR of the model with only the random intercepts for facility with the MORs of models adjusted for different subsets of patient-level variables: only demographics, only preexisting comorbidities, only complicated DFU at presentation, only other social drivers of health (SVI, rurality, and drive time to nearest VHA tertiary hospital), and full model adjusted for all patient-level variables. We conducted 2 sensitivity analyses to calculate the MOR for major leg amputation. First, we excluded veterans who received a major leg amputation within 1 month of DFU diagnosis to exclude those presented too late to consider salvaging the leg. Second, we excluded veterans who died during the follow-up period to determine the extent that MOR was affected by the censoring due to mortality. All statistical tests were 2-sided, and statistical significance was defined as α < .05. All analyses were performed using SAS software, version 9.4 (SAS Institute Inc).

## Results

This cohort included 86 094 veterans (98.3% male and 1.7% female; mean [SD] age, 73.0 [8.1] years; age range, 55-102 years) from 140 VHA facilities who were newly diagnosed with DFU (Table 1). A total of 0.9% were American Indian or Alaska Native; 1.5% were Asian, Native Hawaiian, Pacific Islander, or multicultural; 1.6% were Black; 77.8% were White; and 3.3% had unknown race. In addition, 5.8% of Veterans were Hispanic, 92.7% were not Hispanic, and 1.5% had unknown ethnicity. Nearly 34.3% lived in rural or highly rural areas, and 75.5% had a drive time greater than 30 minutes to their nearest VHA tertiary hospital. A total of 38.2% lived in a neighborhood with a high or very high SVI. The most common comorbidities were hypertension (84.6%), peripheral vascular disease (PVD; 33.1%), and chronic kidney disease (CKD; 30.2%). At presentation, 92.5% had an uncomplicated DFU. Complicated DFU and PVD at presentation were the 2 variables with the largest difference between veterans who had major leg amputation and those who did not.

**Table 1.** Characteristics of 86 094 Patients With New Diagnosis of DFU From 2016 to 2021 by Amputation Status

Characteristic	Patients, No. (%)		Standardized mean difference
	Major leg amputation (n = 3279)	No major leg amputation (n = 82 815)	
Age group, y			
55-65	1149 (35.0)	25 303 (30.6)	3.12
66-75	1589 (48.5)	39 353 (47.5)	0.74
76-85	422 (12.9)	12 978 (15.7)	−1.69
>85	119 (3.6)	5181 (6.3)	−1.51
Sex			
Male	3251 (99.2)	81 383 (98.3)	5.26
Female	28 (0.8)	1432 (1.7)	−0.50
Race			
American Indian or Alaska Native	42 (1.3)	724 (0.9)	0.23
Asian, Native Hawaiian, Pacific Islander, or multiracial	50 (1.5)	1202 (1.4)	0.04
Black	1057 (32.2)	13 225 (16.0)	11.05
White	2023 (61.7)	64 932 (78.4)	−15.29
Unknown	107 (3.3)	2732 (3.3)	−0.02
Ethnicity			
Hispanic or Latino	276 (8.4)	4720 (5.7)	1.60
Not Hispanic or Latino	2961 (90.3)	76 831 (92.8)	−4.48
Unknown	42 (1.3)	1264 (1.5)	−0.14
Residency			
Urban	2363 (71.0)	54 214 (65.5)	5.78
Rural or highly rural	963 (29.0)	28 559 (34.5)	−3.71
Drive time to nearest VHA tertiary hospital, min			
0-30	1196 (36.5)	19 644 (23.7)	8.95
>30	2069 (63.1)	62 934 (76.0)	−12.00
Unknown	14 (0.4)	237 (0.3)	0.08
SVI			
Very high SVI	877 (26.4)	15 720 (19.0)	4.72
High SVI	618 (18.6)	15 639 (19.0)	−0.21
Moderate SVI	536 (16.1)	14 666 (17.7)	−0.93
Low SVI	398 (12.0)	12 507 (15.1)	−1.85
Very low SVI	238 (7.2)	7825 (9.4)	−1.39
Unknown	659 (19.8)	16 416 (19.8)	0.05
Complicated DFU at presentation			
Uncomplicated DFU	2144 (65.4)	77 479 (93.6)	−27.32
Osteomyelitis or gangrene	1135 (34.6)	5336 (6.4)	19.41
Comorbidities			
Chronic kidney disease	1500 (45.8)	24 510 (29.6)	12.24
Congestive heart failure	1200 (36.6)	20 683 (25.0)	8.17
Pulmonary circulation disease	210 (6.4)	3854 (4.6)	1.02
Peripheral vascular disease	2203 (67.2)	26 314 (31.8)	34.03
Hypertension	3000 (91.5)	69 834 (84.3)	13.57
Myocardial infarction	518 (15.8)	5734 (6.9)	5.42
Cerebrovascular disease	728 (22.2)	12 406 (15.0)	4.59
Liver disease	353 (10.8)	6217 (7.5)	1.94
Neurologic disease	463 (14.1)	9121 (11.0)	1.88
Paralysis	108 (3.3)	1963 (2.4)	0.53
Dementia	334 (10.2)	6870 (8.3)	1.12
Chronic obstructive pulmonary disease	886 (27.0)	20 266 (24.5)	1.68
HIV or AIDS	28 (0.8)	356 (0.4)	0.24
Lymphoma	40 (1.2)	1024 (1.2)	−0.01
Solid cancer	434 (13.2)	9689 (11.7)	0.93
Metastatic cancer	45 (1.4)	1197 (1.4)	−0.05
Rheumatoid arthritis	82 (2.5)	2529 (3.0)	−0.31
Obesity	684 (20.9)	21 732 (26.2)	−3.40

Abbreviations: DFU, diabetic foot ulcer; SVI, Social Vulnerability Index; VHA, Veterans Health Administration.

Major leg amputation was performed for 3.8% of patients within 1 year from DFU diagnosis. Among those who had major leg amputation, 73.3% underwent below-knee amputation, and 41.7% received above-knee amputation, whereas 15.0% underwent both below- and above-knee amputation sequentially. A total of 95.1% of veterans underwent amputation within the same facility where they received their DFU diagnosis. Regarding the timing of major leg amputation, 35.9% had major leg amputation within 1 month of DFU diagnosis, and 33.5% had major leg amputation in 1 to 3 months of DFU diagnosis.

Previously identified social drivers of health and other patient-level factors were associated with major leg amputation. Social drivers included very high SVI (very low SVI as a reference; OR, 1.22; 95% CI, 1.04-1.43) and identifying as Black (OR, 1.92; 95% CI, 1.74-2.11), American Indian or Alaska Native (OR, 1.76; 95% CI, 1.26-2.47), or Hispanic (OR, 1.42; 95% CI, 1.21-1.67) (Table 2). Patient-level comorbidities included complicated DFU at presentation (OR, 4.79; 95% CI, 4.41-5.21), PVD (OR, 3.20; 95% CI, 2.95-3.47), myocardial infarction (OR, 1.46; 95% CI, 1.30-1.63), CKD (OR, 1.33; 95% CI, 1.23-1.44), and hypertension (OR, 1.23; 95% CI, 1.08-1.40) (Table 2).

**Table 2.** AORs of Fixed Patient-Level Variables for Major Leg Amputation Within 1 Year of DFU Diagnosis

Characteristic	AOR (95% CI)^a^	*P* value
Age group, y		
55-65	1 [Reference]	NA
66-75	0.89 (0.81-0.96)	.005
76-85	0.67 (0.59-0.76)	<.001
>85	0.47 (0.38-0.57)	.001
Sex		
Male	1 [Reference]	NA
Female	0.55 (0.37-0.82)	.003
Race		
American Indian or Alaska Native	1.76 (1.26-2.47)	.001
Asian, Native Hawaiian, Pacific Islander, or multiracial	1.09 (0.80-1.48)	.58
Black	1.92 (1.74-2.11)	.001
White	1 [Reference]	NA
Unknown	1.25 (0.99-1.57)	.06
Ethnicity		
Hispanic or Latino	1.42 (1.21-1.67)	.001
Not Hispanic or Latino	1 [Reference]	NA
Unknown	0.87 (0.61-1.24)	.45
Residency		
Urban	1 [Reference]	NA
Rural or highly rural	1.02 (0.93-1.12)	.66
Drive time to nearest VHA tertiary hospital, min		
0-30	1 [Reference]	NA
>30	0.88 (0.80-0.97)	.01
Unknown	1.77 (0.97-3.23)	.06
SVI		
Very high SVI	1.22 (1.04-1.43)	.02
High SVI	1.06 (0.90-1.25)	.49
Moderate SVI	1.11 (0.94-1.30)	.24
Low SVI	1.04 (0.88-1.24)	.66
Very low SVI	1 [Reference]	NA
Unknown	1.15 (0.98-1.35)	.10
Complicated DFU at presentation		
Uncomplicated DFU	1 [Reference]	NA
Osteomyelitis or gangrene	4.79 (4.41-5.21)	<.001
Comorbidities		
Chronic kidney disease	1.33 (1.23-1.44)	<.001
Congestive heart failure	1.08 (0.99-1.18)	.08
Pulmonary circulation disease	0.92 (0.79-1.08)	.33
Peripheral vascular disease	3.20 (2.95-3.47)	<.001
Hypertension	1.23 (1.08-1.40)	.002
Myocardial infarction	1.46 (1.30-1.63)	<.001
Cerebrovascular disease	1.10 (0.99-1.21)	.06
Liver disease	1.03 (0.91-1.16)	.66
Neurologic disease	0.97 (0.87-1.09)	.60
Paralysis	1.10 (0.89-1.36)	.36
Dementia	0.94 (0.82-1.07)	.35
Chronic obstructive pulmonary disease	0.95 (0.87-1.03)	.21
HIV or AIDS	1.44 (0.96-2.17)	.08
Lymphoma	0.90 (0.63-1.27)	.53
Solid cancer	1.02 (0.90-1.15)	.77
Metastatic cancer	0.72 (0.52-1.01)	.05
Rheumatoid arthritis	0.82 (0.65-1.03)	.09
Obesity	0.73 (0.67-0.80)	<.001

Abbreviations: AOR, adjusted odds ratio; DFU, diabetic foot ulcer; NA, not applicable; SVI, Social Vulnerability Index; VHA, Veterans Health Administration.

^a^ Adjusted for all other variables included in the table.

The facilities’ ORs for facility-level variation in major leg amputation, adjusted for all social drivers and patient-level comorbidities, ranged from 0.29 (95% CI, 0.12-0.67) to 3.53 (95% CI, 2.57-4.86) (Figure). Table 3 gives the MORs of various models using different sets of patient-level variables. The MOR was 2.26 in the model with only random facility intercepts and decreased as additional patient-level variables were included in the model. The MOR of the full model, which included demographics, comorbidities, complicated DFU at presentation, and other social drivers of health, was 1.85, indicating that the odds of major leg amputation were 1.85 times higher between 2 randomly selected facilities for the average veteran, with the likelihood ratio test showing a significant improvement in the model with the inclusion of random facility intercepts (*P* < .001).

**Figure.**
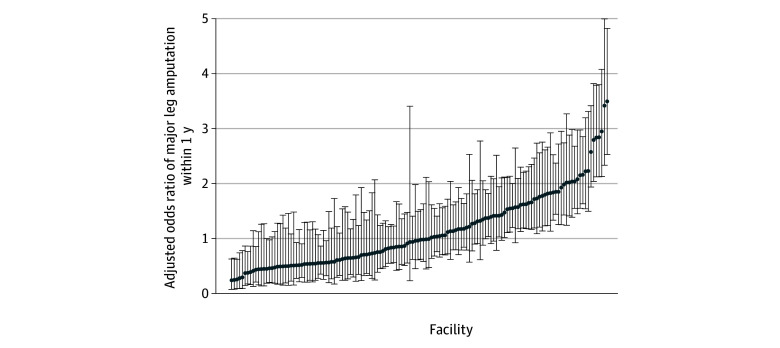
Variation in the Adjusted Odds Ratio of Major Leg Amputation Within 1 Year From Diabetic Foot Ulcer Diagnosis Among 140 Veterans Health Administration Facilities Error bars indicate 95% CIs.

**Table 3.** Change in the MORs for Hospital-Level Variation of Major Lower Limb Amputation Within 1 Year of DFU Diagnosis According to Varying Model of Adjustment for Patient-Level Variables

Model variable	MOR
Only random effect of facility—no fixed patient-level effect	2.26
Adjusted for SVI, drive time to nearest VHA tertiary hospital, and rurality	2.15
Adjusted for demographics (age, sex, race, and ethnicity)	2.13
Adjusted for preexisting comorbidities	2.13
Adjusted for complicated DFU at presentation	2.05
Full model adjusted for all patient-level variables	1.85

Abbreviations: DFU, diabetic foot ulcer; MOR, median odds ratio; SVI, Social Vulnerability Index; VHA, Veterans Health Administration.

A total of 15.6% of the veterans in this cohort died within 1 year of their DFU diagnosis. The results of the multivariable mixed-effects regression model estimating 1-year mortality are given in eTable 2 in Supplement 1. The ORs for facility-level variation in 1-year mortality, adjusted for social drivers and patient-level comorbidities, ranged from 0.69 (95% CI, 0.56-0.85) to 1.42 (95% CI, 1.20-1.68) (eFigure in Supplement 1). The MOR was 1.16 (*P* < .001).

In the sensitivity analysis excluding veterans who received a major leg amputation within 1 month of their DFU diagnosis (n = 84 916), the MOR of the full model was 1.69 (*P* < .001). In another sensitivity analysis using a cohort excluding veterans who died (n = 72 691), the MOR of the full model was 1.80 (*P* < .001), which was close to that obtained in the full cohort.

## Discussion

In this retrospective study of veterans with newly diagnosed DFU during 2016 to 2021, we found significant facility-level variation in major leg amputation within 1 year of DFU diagnosis, even after controlling for social drivers and patient-level comorbidities. The magnitude of the facility-level MOR (1.85) was larger than the point estimate ORs for many patient-level variables. This suggests that the facilities caring for patients with DFU are important determinants of the likelihood of major leg amputation.

We hypothesize that variation in major leg amputation is largely due to health care system factors related to organization of care in patients with DFUs in each facility because we found a much smaller degree of facility-level variation in all-cause mortality and prior studies^28,29,30,31,32,33,34,35^ report associations between DFU-specific care and limb salvage. Although the sensitive analysis excluding veterans who had a major leg amputation within 1 month of DFU diagnosis suggested that delayed presentation may partly explain the facility-level variation, we believe health care system factors were the main drivers for the variation. These health care system factors include access to primary care and appropriate specialty care, degree of coordination across specialties and settings (including presence of multidisciplinary teams), and local health care policies.^14,36^ They also include treating specific physiologic components, such as glycemic control, guideline-concordant PVD management revascularization, and limb-salvaging operations (incision and drainage surgery or minor leg amputation [leg amputation below the ankle]).^28,29,30,31,32,33,34,35^

As a next step, we plan to assess how facility-level variation in major leg amputation rates is influenced by health care organization and care processes. Facilities serving communities with high rates of social deprivation and excelling at limb salvage may offer insights into closing longstanding disparities. The VHA’s national program PAVE (Prevention of Amputation in Veterans Everywhere) provides an infrastructure to distribute best practices to clinicians vested in limb salvage at each facility, including identification of patients at risk for leg amputation and timely referral of the highest-risk patients to podiatrists.^37^

Consistent with findings from non-VHA studies, we also observed substantial variation in the likelihood of major leg amputation associated with social drivers of health, including race and ethnicity and residence in areas with a high SVI.^5,13,38^ This finding is despite the VA’s minimal barriers to care compared with the general US population and speaks to the pervasive effects of these drivers, which might be blunted but not completely overcome by improved access to insurance and care.

Comorbidities such as PVD, CKD, and myocardial infarction, which have been associated with major leg amputation in previous studies, were also found to be significant in our analyses.^12,39^ It is well known that PVD and decreased perfusion can lead to poor wound healing and gangrene; therefore, it is plausible that PVD is associated with major leg amputation.^5^ Myocardial infarction and CKD are also considered macrovascular complications of diabetes, so it is unsurprising that they are associated with DFU and major leg amputation.

### Limitations

There are several limitations to our study. First, this study used *ICD-9*, *ICD-10*, and *CPT* codes to identify patients with DFU, the severity at presentation, comorbidities, and major leg amputations. Although these codes have been validated in previous studies, miscoding is still possible. Second, our study only included patients with DFU diagnosed within the VHA; veterans with Medicare or other coverage who received care outside the VHA may not have been captured in our study. Moreover, only major leg amputations performed within the VHA system were identified. Veterans may have been referred to a non-VHA hospital for the amputation, leading to potential undercounting of this outcome. Third, we were unable to capture certain patient-level factors that could influence outcomes, such as smoking status. In addition, characteristics of wounds, such as wound size or extent of wound infection, were not readily available without manual medical record review. However, we included a comprehensive set of measurable patient-level factors known to be associated with major leg amputation. Fourth, we used 1 year as a follow-up period to capture outcomes (major leg amputation or mortality). Although a longer follow-up period might identify delayed major leg amputations, we believe 1 year is optimal for capturing early major leg amputations, which are most affected by the variation in multidisciplinary DFU care. Fifth, our analysis for major leg amputation was subject to bias due to censoring from mortality. Nevertheless, we believe the effect of that bias is small because our sensitivity analysis excluding those who died during follow-up showed a similar MOR to that from the original cohort. Sixth, we only adjusted for patient-level social drivers of health in this cohort. However, facilities serving communities with high burdens of social marginalization may have additional constraints related to resources on their ability to provide adequate care. This limitation may have led to underadjustment for social drivers when measuring at the patient rather than community level.

## Conclusions

In this VHA-wide cohort study of veterans with a new diagnosis of DFU, we found significant facility-level variation in major leg amputation rates within 1 year of DFU diagnosis. Facility-level variation in 1-year mortality rates was much smaller, suggesting variation in leg amputation was likely to stem from variation in DFU-specific care. The VHA should strive to both minimize the odds of major leg amputation and interfacility variation. One promising way to do this is to identify and disseminate particularly effective care processes, including organization of care.

## Notes

Supplement 1. **eTable 1.** Definitions for Variables **eTable 2.** Adjusted Odds Ratios of Fixed Patient-Level Variables for All-Cause Mortality Within 1 Year of Diabetic Foot Ulcer Diagnosis **eFigure.** Variation in the Adjusted Odds Ratio of Mortality Within 1 Year of Diabetic Foot Ulcer Diagnosis Among 140 Veterans Health Administration Facilities

Supplement 2.
Data Sharing Statement
