# Supplementary material for: Facility-Level Variation in Major Leg Amputation Among Patients With Newly Diagnosed Diabetic Foot Ulcer
Source: JAMA Netw Open. 2025 Apr 23;8(4):e256781. doi: 10.1001/jamanetworkopen.2025.6781 (PMC12019509; doi:10.1001/jamanetworkopen.2025.6781)
Supplement: Supplement 1. — eTable 1. Definitions for Variables eTable 2. Adjusted Odds Ratios of Fixed Patient-Level Variables for All-Cause Mortality Within 1 Year of Diabetic Foot Ulcer Diagnosis eFigure. Variation in the Adjusted Odds Ratio of Mortality Within 1 Year of Diabetic Foot Ulcer Diagnosis Among 140 Veterans Health Administration Facilities [file jamanetwopen-e256781-s001.pdf]

Supplemental Online Content

Suzuki H, Vaughan-Sarrazin M, Ohl M, et al. Facility-level variation in major leg amputation among patients with newly diagnosed diabetic foot ulcer. *JAMA Netw Open*. 2025;8(4):e256781. doi:10.1001/jamanetworkopen.2025.6781

eTable 1. Definitions for Variables

eTable 2. Adjusted Odds Ratios of Fixed Patient-Level Variables for All-Cause Mortality Within 1 Year of Diabetic Foot Ulcer Diagnosis

eFigure. Variation in the Adjusted Odds Ratio of Mortality Within 1 Year of Diabetic Foot Ulcer Diagnosis Among 140 Veterans Health Administration Facilities

This supplemental material has been provided by the authors to give readers additional information about their work.

© 2025 Suzuki H et al. *JAMA Network Open*.

eTable 1. Definitions for Variables

Variables	
Diabetic foot ulcer	ICD-10: (E08.621, E09.621, E10.621, E11.621, E13.621)<br>OR (L97.3-L97.5 plus E08-E013)
Osteomyelitis	ICD-10: 3-L97.304, L97.306, L97.314, L97.316, L97.324, L97.326, L97.404, L97.406, L97.414, L97.416, L97.424, L97.426, L97.504, L97.506, L97.514, L97.516, L97.524, L97.526, M86.17, M86.27, M86.37, M86.47, M86.67, M86.8X7
Gangrene	ICD-10: (E08.52, E09.52, E10.52, E11.52, E13.52)<br>OR ((A48.0, I70.269, I96) plus (E08.51, E09.51, E10.51, E11.51, E13.51))
Major leg amputation	Below knee amputation<br>ICD-10: 0Y6H0Z1, 0Y6H0Z2, 0Y6H0Z3, 0Y6J0Z1, 0Y6J0Z2, 0Y6J0Z3, 0Y6M0Z0, 0Y6N0Z0<br>CPT: 27880, 27881, 27882, 27886, 27888, 27889<br>Above knee amputation<br>ICD-10: 0Y670ZZ, 0Y680ZZ, 0Y6C0Z1, 0Y6C0Z2, 0Y6C0Z3, 0Y6D0Z1, 0Y6D0Z2, 0Y6D0Z3, 0Y6F0ZZ, 0Y6G0ZZ<br>CPT: 27295, 27590, 27591, 27592, 27596, 27598

© 2025 Suzuki H et al. *JAMA Network Open*.

eTable 2. Adjusted Odds Ratios of Fixed Patient-Level Variables for All-Cause Mortality Within 1 Year of Diabetic Foot Ulcer Diagnosis

Variables	Adjusted OR (95% CI)	P-value
**Age**		
55-65	Reference	
66-75	2.00 (1.89-2.12)	<.001
76-85	3.62 (3.39-3.97)	<.001
>85	6.52 (6.01-7.07)	<.001
**Gender**		
Male	Reference	
Female	0.72 (0.59-0.88)	.001
**Race**		
Asian, Native Hawaiian, Pacific Islander, Multiracial	1.06 (0.89-1.25)	.51
Black	0.88 (0.83-0.94)	<.001
Native American	0.85 (0.67-1.08)	.18
White	Reference	
Unknown	1.31 (1.16-1.47)	<.001
**Ethnicity**		
Hispanic or Latino	0.96 (0.87-1.06)	.40
Not Hispanic or Latino	Reference	
Unknown	1.24 (1.04-1.47)	.01
**Residency**		
Urban	Reference	
Rural or highly rural	1.00 (0.95-1.05)	.88
**Drive time to parental tertiary hospital**		
0-30 min	Reference	
>30 min	1.07 (1.01-1.13)	.02
Unknown	1.22 (0.83-1.79)	.31
**Social vulnerability index**		
Very high SVI	1.14 (1.05-1.24)	.001
High SVI	1.07 (0.99-1.16)	.11
Moderate SVI	1.06 (0.98-1.15)	.16
Low SVI	1.03 (0.95-1.12)	.50
Very low SVI	Reference	
Unknown	0.97 (0.90-1.06)	.53
**Complicated DFU at presentation**		
Uncomplicated DFU	Reference	
Osteomyelitis/gangrene	1.46 (1.36-1.56)	<.001

© 2025 Suzuki H et al. *JAMA Network Open*.

Variables	Adjusted OR (95% CI)	P-value
**Comorbidities**		
Chronic kidney disease	1.82 (1.74-1.90)	<.001
Congestive heart failure	2.05 (1.96-2.15)	<.001
Pulmonary circulation disease	1.37 (1.26-1.48)	<.001
Peripheral vascular disease	1.40 (1.34-1.46)	<.001
Hypertension	0.78 (0.74-0.83)	<.001
Myocardial infarction	1.40 (1.31-1.50)	<.001
Cerebrovascular disease	1.17 (1.11-1.23)	<.001
Liver disease	1.46 (1.36-1.57)	<.001
Neurological disease	1.34 (1.27-1.42)	<.001
Paralysis	1.24 (1.09-1.40)	.001
Dementia	2.27 (2.14-2.42)	<.001
Chronic obstructive pulmonary disease	1.29 (1.24-1.35)	<.001
HIV/AIDS	1.05 (0.76-1.44)	.77
Lymphoma	1.26 (1.07-1.47)	.006
Solid cancer	1.27 (1.19-1.35)	<.001
Metastatic cancer	3.66 (3.20-4.18)	<.001
Rheumatoid arthritis	1.16 (1.04-1.30)	.007
Obesity	0.71 (0.68-0.75)	<.001

OR: Odds Ratio, CI: Confidence Interval, VHA: Veterans Health Administration, SVI: Social Vulnerability Index, DFU: Diabetic foot ulcer, HIV: Human Immunodeficiency Virus, AIDS: Acquired Immunodeficiency Syndrome

© 2025 Suzuki H et al. *JAMA Network Open*.

eFigure. Variation in the Adjusted Odds Ratio of Mortality Within 1 Year of Diabetic Foot Ulcer Diagnosis Among 140 Veterans Health Administration Facilities

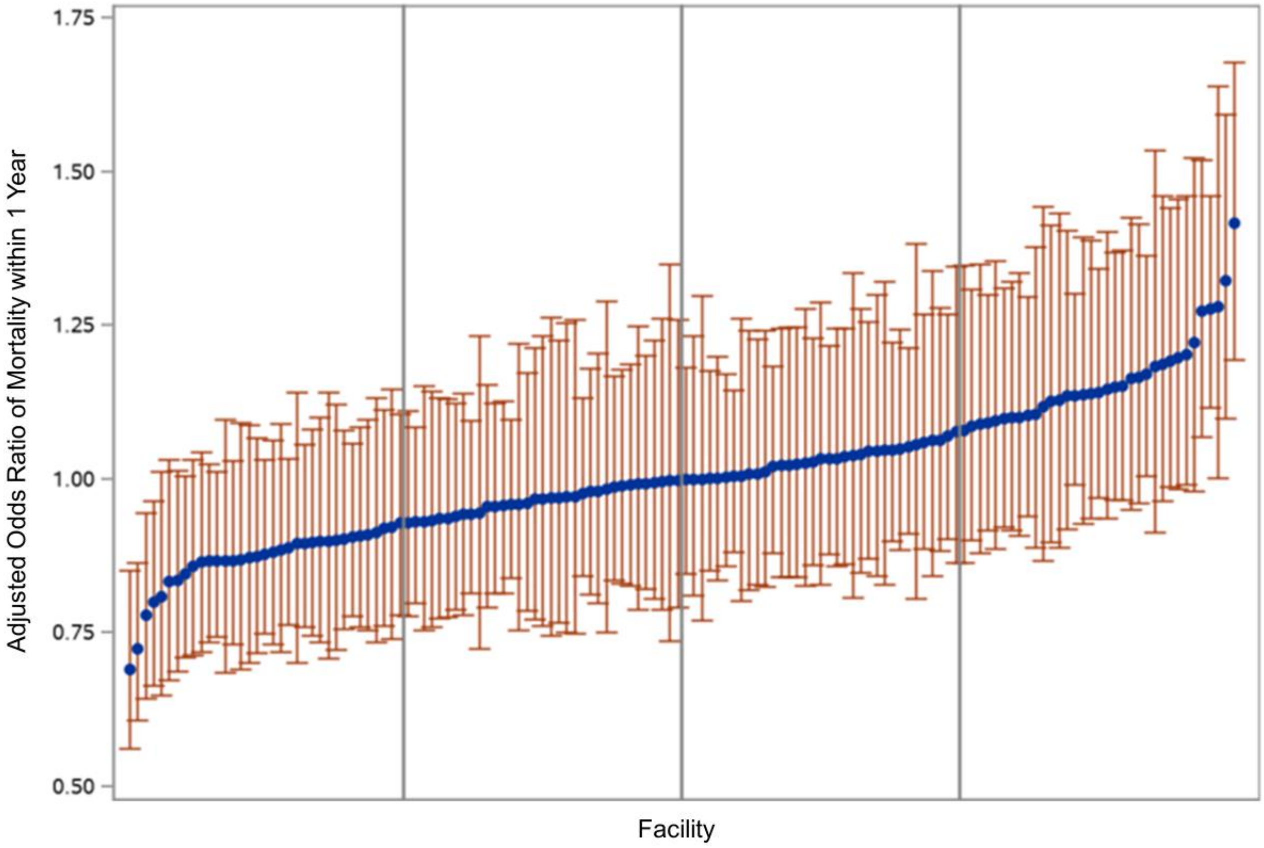

© 2025 Suzuki H et al. *JAMA Network Open*.
