# Supplementary material for: Facility-Level Variation in Major Leg Amputation Among Patients With Newly Diagnosed Diabetic Foot Ulcer
Source: JAMA Netw Open. 2025 Apr 23;8(4):e256781. doi: 10.1001/jamanetworkopen.2025.6781 (PMC12019509; doi:10.1001/jamanetworkopen.2025.6781)
Supplement: Supplement 2. — Data Sharing Statement [file jamanetwopen-e256781-s002.pdf]

Data Sharing Statement

Suzuki. Facility-Level Variation in Major Leg Amputation Among Patients With Newly Diagnosed Diabetic Foot Ulcer. *JAMA Netw Open*. Published April 23, 2025.
doi:10.1001/jamanetworkopen.2025.6781

Data
Data available: No
